# Complete Blood Count-Based Biomarkers as Predictors of Clinical Outcomes in Advanced Non-Small Cell Lung Cancer Patients with PD-L1 < 50% Treated with First-Line Chemoimmunotherapy

**DOI:** 10.3390/curroncol31090367

**Published:** 2024-08-26

**Authors:** Carlo Putzu, Riccardo Serra, Rachele Campus, Giovanni Maria Fadda, Claudio Sini, Andrea Marongiu, Giorgio Carlo Ginesu, Alessandro Giuseppe Fois, Giuseppe Palmieri, Angelo Zinellu, Antonio Cossu, Panagiotis Paliogiannis

**Affiliations:** 1 Medical Oncology Unit, University Hospital of Sassari (AOU SS), Via Enrico De Nicola 39, 07100 Sassari, Italygiovanni.fadda@aouss.it (G.M.F.); 2 Specialty School of Medical Oncology, University of Cagliari, S.S. 554, Km 4500 Bivio per Sestu, 09042 Cagliari, Italy; 3 Specialty School in Pulmonology and Respiratory Diseases, University of Sassari, Viale San Pietro 43a, 07100 Sassari, Italy; 4 Medical Oncology Unit, Giovanni Paolo II Hospital of Olbia, Via Bazzoni Sircana 1, 07026 Olbia, Italy; 5 Department of Medicine, Surgery and Pharmacology, University of Sassari, Viale San Pietro 43a, 07100 Sassari, Italyginesugc@uniss.it (G.C.G.);; 6 Department of Biomedical Sciences, University of Sassari, Viale San Pietro 43a, 07100 Sassari, Italy; gpalmieri@uniss.it (G.P.);

**Keywords:** lung cancer, chemotherapy, immunotherapy, NLR, MNR, biomarkers, blood cell count

## Abstract

**Background:** The aim of the study was to investigate a series of complete blood cell count-based biomarkers of systemic inflammation as predictors of clinical outcomes in patients who underwent first-line chemoimmunotherapy for advanced NSCLC. **Methods:** Consecutive patients with pathologically diagnosed stage III/IV NSCLC and PD-L1 < 50% who underwent first-line chemoimmunotherapy were retrospectively enrolled. The clinical outcomes used for biomarker evaluation were Objective Response Rate (ORR) and Overall Survival (OS). **Results:** Non-responders had significantly higher values of neutrophil to lymphocyte ratio (NLR, median: 5.36; IQR: 2.78–10.82 vs. 3.31; IQR: 2.15–4.12, *p* = 0.019), neutrophil to monocyte ratio (NMR, median: 14.00; IQR: 8.82–21.20 vs. 9.20; IQR: 7.45–11.20, *p* = 0.013), and systemic inflammation index (SII, median: 1395; IQR: 929–3334 vs. 945; IQR: 552–1373, *p* = 0.025), but only NLR and NMR remained independently associated with clinical response in multivariate logistic regression. In the univariate analysis, white blood cells (OR:1.2202; 95% CI: 1.0339–1.4400, *p* = 0.019), neutrophils (OR:1.2916; 95% CI: 1.0692–1.5604, *p* = 0.008), NLR (OR:1.3601: 95% CI: 1.0949–1.6896, *p* = 0.005) and NMR (OR:1.2159; 95% CI: 1.00396–1.4221, *p* = 0.015) were significantly associated with survival; Cox regression models confirmed that neutrophils, NLR, and MLR were independently associated with survival; NLR, at a cut-off value of 4.0, showed the better AUC (0.749) in predicting OS. **Conclusions:** Baseline complete blood cell count biomarkers, especially the NLR, can predict clinical outcomes in patients with advanced NSCLC treated with first-line chemoimmunotherapy.

## 1. Introduction

Lung cancer is the most common type of cancer worldwide [1,2]. Despite significant progresses in identifying and treating the disease, mortality rates remain very high, closely matching the rates of incidence [1]. This is due to several factors, including the subtle onset of the disease, leading to delays in clinical evaluation and diagnosis, the poor understanding of the underlying pathogenic mechanisms, and the lack of effective treatments, especially for patients in the advanced disease stage. A wider implementation of low-dose-computed tomography lung cancer screening for specific at-risk population groups worldwide could reduce mortality rates, by detecting the disease at an earlier stage [3].

In advanced NSCLC stages, the systemic therapies are currently divided into two different frontline therapeutic approaches, depending on whether the disease is oncogene- or non-oncogene-addicted [4]. In oncogene-addicted cases, specific targeted therapies against the precise genetic alteration detected are used. In non-oncogene-addicted disease, different immunotherapy strategies with various immune check point inhibitors (ICIs), depending on the values of the immunohistochemical expression of the biomarker programmed death ligand 1 (PD-L1), can be used. For PD-L1 expression values greater than 50%, single-agent immunotherapy is recommended; for PD-L1 values 0–49% a combination of a chemotherapy agent with an ICI is suggested [5,6]. PD-L1 is, therefore, the only biomarker currently validated for the selection of immunotherapy treatment strategies in advanced stage NSCLC patients with non-oncogene-addicted disease [7]. Nevertheless, the immunohistochemical evaluation of PD-L1 has several technical limitations, and no alternative biomarkers are currently available for an accurate prediction of the clinical outcomes of chemoimmunotherapy treatments. 

Complete blood count-based indexes of systemic inflammation, have been shown to have interesting predictive roles in several lung diseases, including lung cancer [8,9,10,11,12]. In particular, the neutrophil to lymphocyte ratio (NLR) showed good performances in predicting clinical outcomes after both surgical and medical treatments of patients with NSCLC [13,14,15]. Numerous studies investigated the prognostic and predictive roles of systemic blood count-based inflammation biomarkers in patients submitted to first-line immunotherapy, with a single ICI [13,16,17]. However, limited data are available regarding their role in patients with NSCLC treated with first-line chemoimmunotherapy [17]. For this reason, we conducted a retrospective study to assess the association between clinical response and overall survival (OS) and the most common blood-based inflammation biomarkers like NLR, platelet to lymphocyte ratio (PLR), neutrophil to monocyte ratio (NMR), monocyte to lymphocyte ratio (MLR), the systemic inflammation index (SII), and the aggregate index of systemic inflammation (AISI), in patients with advanced-stage NSCLC and PD-L1 tumor proportion score (TPS) lower than 50% who underwent first-line chemoimmunotherapy.

## 2. Materials and Methods

### 2.1. Patients and Clinical Data

Consecutive patients with pathologically diagnosed stage III/IV NSCLC treated in the medical oncology units of the University Hospital of Sassari and the Hospital of Olbia from January 2019 to December 2023 were retrospectively enrolled in this real-world observational study. The inclusion criteria were as follows: (1) age 18 years or older; (2) affected by histologically and/or immunohistochemically diagnosed NSCLC; (3) advanced stage disease (III or IV stage) at diagnosis; (4) PD-L1 TPS between 0 and 49; (5) no somatic mutations in the EGFR, KRAS or BRAF genes through Next Generation Sequencing (Myriapod^®^ NGS Cancer panel DNA), and no fusions in the ALK, ROS1, MET, RET and NTRK genes through real-time PCR (IDYLLA™ Gene Fusion Assay) detected; (6) patients with at least 6 months of follow-up from treatment initiation; (7) patients with available clinical, pathological and laboratory data; and (8) patients who signed the informed consent for the medical procedures performed and for their participation into the study. The demographic and clinical data recorded, included information on gender, smoking status, Eastern Cooperative Oncology Group (ECOG) Performance Status, clinical stage as per American Joint Committee on Cancer (AJCC) 8th edition, and comorbidities; oncological response assessment was performed according to the Response Evaluation Criteria in Solid Tumors version 1.1 (RECIST 1.1) by computed tomography scans every 6 to 12 weeks after treatment initiation. The clinical outcomes used for biomarker evaluation were Objective Response Rate (ORR) and Overall Survival (OS). The clinical, pathological and follow-up data were registered in a dedicated digital database, exclusively accessible by the researchers participating into the study. The study was performed in accordance with the principles of the Declaration of Helsinki on human research and was approved by the local Ethics Committee (BIOSURG-SS; PROT. PG/2019/4493). 

### 2.2. Laboratory Tests

Routine laboratory tests performed before treatment initiation were evaluated. Fasting blood samples were obtained following standard procedures and protocols by current international and national guidelines, and were analyzed in a certified laboratory. Basic blood test parameters like hemoglobin, red cell distribution width (RDW), the number of platelets, and numbers of inflammation cell populations were investigated as predictors of the clinical outcomes mentioned above. In addition, the NLR, MLR, and PLR were tested, as well as the SII (neutrophils × platelets divided by the number of lymphocytes), and the AISI (neutrophils × monocytes × platelets divided by the number of lymphocytes). 

### 2.3. Statistical Analysis

To evaluate the distribution of the variables, the Kolmogorov–Smirnov was used, and data were expressed as mean (mean ± SD) or median values (median and IQR). The differences in continuous variables between the groups were compared using unpaired Student’s *t*-test or Mann–Whitney rank sum test, as appropriate. The differences between categorical variables were evaluated using the chi-squared test. A receiver-operating characteristics (ROC) curve analysis was performed to define the ideal cut-off values to maximize sensitivity and specificity, according to the Youden index. The association between variables and outcomes was evaluated by univariate and multivariate logistic regression. To avoid overfit, two different models were proposed for multivariate analysis. Model 1 with correction for age, gender, smoking status, histological type, and PD-L1, and Model 2 with correction for age, gender, smoking status, stage T, and stage N. In addition, to reduce the risk of collinearity bias, the independent diagnostic power of different hematological parameters was separately assessed in the models. To evaluate survival, the time of diagnosis was set as time zero. The survival probability was estimated using Kaplan–Meier curves, assuming death as end point. Cox proportional hazards regression was performed for both univariate and multivariate analyses. For multivariate Cox regression, the same models proposed above have been utilized as well. Statistical analyses were performed using MedCalc for Windows, version 22.0.21, 64 bit (MedCalc Software, Ostend, Belgium).

## 3. Results

### 3.1. Blood-Based Biomarkers and Treatment Response

A total of 62 patients [45 males and 17 females; median age at diagnosis, 68.5 (IQR: 62.0–74.0) years] were included into the study (Table 1). Forty-seven patients (75.8%) were responders, while the remaining fifteen (24.2%) were non-responders. Responders, as expected, showed a significantly decreased mortality rate (22% vs. 80%, *p* < 0.0001), and increased OS: median 14.5 months (IQR: 9.1–31.9 months) vs. 7.5 months (IQR 4.2–11.2 months) in non-responders, *p* = 0.0015. Non-responders had significantly higher values of NLR (median: 5.36; IQR: 2.78–10.82 vs. 3.31; IQR: 2.15–4.12, *p* = 0.019), NMR (median: 14.00; IQR: 8.82–21.20 vs. 9.20; IQR: 7.45–11.20, *p* = 0.013), and SII (median: 1395; IQR: 929–3334 vs. 945; IQR: 552–1373, *p* = 0.025). There were no statistically significant differences between responders and non-responders in age, gender, smoking status, histological type, PD-L1 expression, T stage, N stage, Hb, RDW, WBC, neutrophils, lymphocytes, monocytes, platelets, MLR, PLR, and AISI.

In the univariate logistic regression analysis, only NLR (OR = 1.2561, 95% CI 1.0519 to 1.4998, *p* = 0.012) and NMR (OR = 1.1410, 95% CI 1.0121 to 1.2864, *p* = 0.03) were significantly associated with treatment response (Table 2). 

These results were also confirmed by multivariate logistic regression. As reported in Table 3, two distinct models were employed to mitigate the risk of overfitting. The first one (Model 1) included age, gender, smoking status, histological type, and PD-L1 as confounders, whereas the second model (Model 2) comprised age, gender, smoking status, stage T, and stage N. In both models, NLR and NMR remained independently related with the clinical response to the treatment.

ROC curve analysis was performed to evaluate the sensitivity, specificity, and accuracy of NLR and NMR in identifying responders from non-responders. The area under the curve (AUC) values were 0.703 (95% CI 0.573 to 0.812) for NLR and 0.715 (9%% CI 0.586 to 0.822) for NMR. The sensitivity and specificity at a NLR cut-off value of 4.56 were 60% and 81%, respectively, while for an NMR cut-off value of 13.7, they were 53% and 91%, respectively.

### 3.2. Blood-Based Biomarkers and Overall Survival

Data regarding OS were available for 60 patients [44 males and 16 females; median age at diagnosis, 68.0 (IQR: 62.0–73.5) years] (Table 4). Thirty-seven patients (61.7%) were alive, while the remaining 23 (28.3%) died. Non-survivors had significantly higher values of WBC (median: 9.69 × 10^9^ L; IQR: 7.97–14.03 × 10^9^ L vs. 8.26 × 10^9^ L IQR: 6.36–9.92 × 10^9^ L, *p* = 0.026), neutrophils (median: 7.00 × 10^9^ L; IQR: 6.00–11.95 × 10^9^ L vs. 5.30 × 10^9^ L IQR: 3.48–7.03 × 10^9^ L, *p* = 0.001), NLR (median: 4.56; IQR: 3.07–9.49 vs. 2.94; IQR: 1.92–3.88, *p* = 0.012), NMR (median: 10.40; IQR: 8.80–18.18 vs. 9.00; IQR: 7.08–11.05, *p* = 0.007), PLR (median: 220; IQR: 145–273 vs. 136; IQR: 107–201, *p* = 0.016), SII (median: 1493; IQR: 1000–2578 vs. 849; IQR: 488–1081, *p* = 0.0004), and AISI (median: 1016; IQR: 470–1836 vs. 351; IQR: 256–794, *p* = 0.006). There were no significant differences between survivors and non-survivors in age, gender, smoking status, histological type, PD-L1, T stage, N stage, Hb, RDW, lymphocytes, monocytes, platelets, and MLR.

In the univariate logistic regression analysis, only WBC (OR = 1.2202, 95% CI 1.0339 to 1.4400, *p* = 0.019), neutrophils (OR = 1.2916, 95% CI 1.0692 to 1.5604, *p* = 0.008), NLR (OR = 1.3601, 95% CI 1.0949 to 1.6896, *p* = 0.005), and NMR (OR = 1.2159, 95% CI 1.0396 to 1.4221, *p* = 0.015) were significantly associated with survival (Table 5). These associations have also been confirmed in multivariate logistic regression after correction for several confounders, using both Models 1 and 2 cited above (Table 6).

With respect to survival, the optimal cutoff values identified by ROC analysis were as follows: WBC, 11.98; neutrophils, 5.7; NLR, 4.0; NMR, 11.8 (Table 6). The values of AUC were 0.672 (0.539 to 0.788) for WBC, 0.746 (0.617 to 0.849) for neutrophils, 0.749 (0.620 to 0.852) for NLR, and 0.707 (0.576 to 0.818) for NMR (Table 7). 

**Figure 1:**
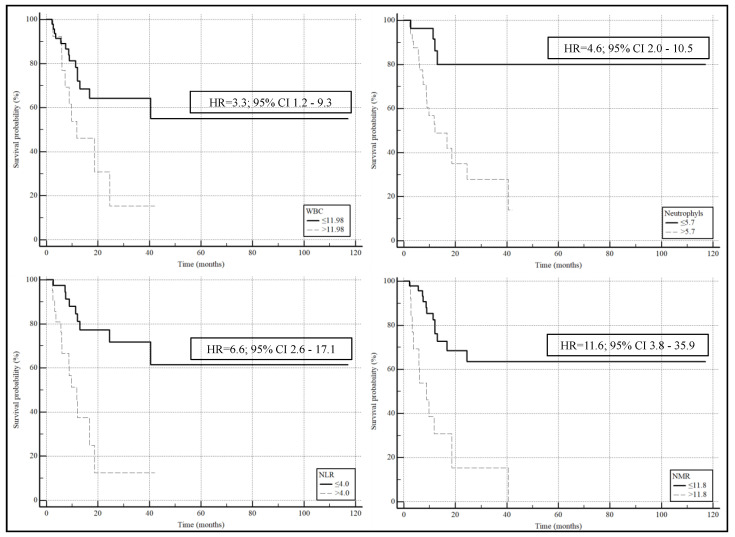
Kaplan–Meier survival curves show significant lower survival with higher values of WBC, neutrophils, NLR, and MLR.

The Kaplan–Meier survival curves, after classifying the patients on the basis of the Youden cut-offs obtained by ROC curves (Figure 1), showed significantly lower survival rates with higher values of WBC (HR= 3.3; 95% CI 1.2–9.3, *p* = 0.02), neutrophils (HR = 4.6; 95% CI 2.00–10.5, *p* = 0.0003), NLR (HR = 6.6; 95% CI 2.6–17.1, *p* = 0.0001), and MLR (HR = 11.6; 95% CI 3.8–35.9, *p* < 0.0001). 

The multivariate Cox regression models reported in Table 8 showed that, excluding WBC, which lost significance in Model 2, both neutrophils, NLR, and MLR were significantly associated with survival after correction for confounders in both models.

## 4. Discussion

The introduction of immunotherapy in clinical practice for the treatment of non-oncogene-addicted NSCLC was one of the most important innovations in medical oncology in the last decade. Several clinical trials have established the efficacy of immune checkpoint blockade, particularly as anti-programmed death 1 (PD-1) antibodies, anti-cytotoxic T-lymphocyte-associated protein 4 (CTLA-4) antibodies, and anti-programmed death 1 ligand (PD-L1) antibodies [18]. Nivolumab, the first monoclonal antibody against PD-1, demonstrated its utility in the treatment of NSCLC and other solid malignancies. Subsequently, pembrolizumab, the second anti-PD-1 antibody, has shown curative effects in several solid cancers, including NSCLC, and consequently other immunotherapy medications have been introduced in clinical practice. Currently, international guidelines suggest the use of first-line monoimmunotherapy when molecular drivers such as EGFR, ALK, ROS1, RET are wild type and the immunohistochemically evaluated PD-L1 TPS is greater than 50%; for patients with lower PD-L1 scores, a combination of chemotherapy and immunotherapy is recommended [4]. 

The addition of chemotherapy to ICIs aims to enhance immune response against cancer through the increase in neoantigen presentation after cancer cell chemo-destruction. In clinical trials, this combination demonstrated significant antineoplastic activity; chemoimmunotherapy regimens that can be used for advanced NSCLC include pembrolizumab plus a platinum-based drug plus pemetrexed [19], pembolizumab plus carboplatin plus paclitaxel or nab-paclitaxel, and others [20]. All these combinations have shown significant improvements in progression-free survival (PFS) and OS when compared to chemotherapy alone. In the KEYNOTE-189 trial, the median OS in the chemoimmunotherapy group was 22.0 months, more than two-fold the median OS of 10.7 months observed in the group of chemotherapy alone [21].

More recently, chemoimmunotherapy with the anti-CTLA4 antibody ipilimumab + the anti-PD-1 nivolumab and two cycles of chemotherapy has been approved for clinical use, based on the results of the CheckMate-9LA trials (*n* = 224 squamous-cell carcinoma patients) [22]. The OS benefit was greater in patients with squamous cell carcinoma (HR 0.63 for squamous cell carcinoma and 0.78 for non-squamous non-small-cell carcinoma) and in those with lack of PD-L1 expression [23]. The introduction of these treatment schemes in clinical practice markedly increased the prognosis for advanced NSCLC patients; nevertheless, only a proportion of patients effectively respond to chemoimmunotherapy and/or experience a durable clinical benefit [24]. For this reason, it is necessary to identify prognostic biomarkers and indicators to predict effective clinical responses and allow time-effective treatment-shifting decisions.

Systemic inflammation in cancer is closely related to and influenced by the activity of both the tumors and the immune system. Tumor-infiltrating lymphocytes (TILs) were found to be strictly bound to the efficacy of immune-based treatments [25], while neutrophils release vascular endothelial growth factor (VEGF), matrix metalloproteinase 9 (MMP-9), and other cytokines which impact the compositions and molecular interactions of the microenvironment of tumors [26,27], while platelets interact with cancer cells, protecting them from immune surveillance [28]. Therefore, circulating indexes of systemic inflammation can be interesting indicators of the complex interaction between cancers and immune responses, as well as of the clinical reflection of such interactions in terms of response to treatments and final survival. Indeed, numerous studies showed that NLR is a good predictor of response to single-agent immunotherapy [13,16,17]. 

Nevertheless, less is known about the usefulness of these indexes in the context of patients submitted to chemoimmunotherapy. Our study showed that non-responders had significantly higher values of NLR (median: 5.36; IQR: 2.78–10.82 vs. 3.31; IQR: 2.15–4.12, *p* = 0.019), NMR (median: 14.00; IQR: 8.82–21.20 vs. 9.20; IQR: 7.45–11.20, *p* = 0.013), and SII (median: 1395; IQR: 929–3334 vs. 945; IQR: 552–1373, *p* = 0.025), but only NLR and NMR remained independently related with the clinical response to the treatment in the multivariate logistic regression models created. In addition, in the univariate logistic regression analysis, we found that only WBC (OR:1.2202; 95% CI: 1.0339–1.4400, *p* = 0.019), neutrophils (OR:1.2916; 95% CI: 1.0692–1.5604, *p* = 0.008), NLR (OR:1.3601: 95% CI: 1.0949–1.6896, *p* = 0.005) and NMR (OR:1.2159; 95% CI: 1.00396–1.4221, *p* = 0.015) were significantly associated with survival. The Cox regression models constructed confirmed that neutrophils, NLR, and MLR were independently associated with survival; NLR, at a cut-off value of 4.0, showed the better AUC (0.749) in predicting survival. 

Shi et al. published a retrospective study in 2021 evaluating potential correlations between peripheral blood biomarkers and clinical outcomes in advanced NSCLC patients who received immunotherapy-based treatments [29]; among the 103 patients enrolled, 71 (68.9%) were treated with chemoimmunotherapy (53 of them as a first-line treatment). In this study, high NLR (>5; log rank *p* = 0.013) and a high PLR (>median of 196.32; log rank *p* = 0.025) were associated with lower OS, but not with the occurrence of adverse therapeutic events in the global cohort; SIRI, SII and AISI were not tested. Focusing on the patients who underwent chemoimmunotherapy, high PLR (HR 3.594, 95% CI: 1.096 to 11.789, *p* = 0.035) was found to be an independent poor prognostic factor for PFS, but not for OS in multivariate analysis. In other words, in this study no correlations between circulating biomarkers of systemic inflammation and survival were found, as in our study. Interestingly, the authors found that the pretreatment absolute lymphocyte count was related to an increased risk of immune-related adverse events in the whole population of the study (OR: 2.165; 95% CI: 1.040–4.509, *p* = 0.039) and patients receiving only ICIs (OR, 6.461; 95% CI: 1.067–39.112; *p* = 0.042), but not in patients who received chemoimmunotherapy [29]. 

More recently, a Spanish multicenter retrospective study was published, including 122 and 92 stage I-IIIB NSCLC patients who received neoadjuvant chemoimmunotherapy followed by surgery as the discovery and the external validation cohorts, respectively [30]. In both the discovery and validation cohorts, the on-treatment NLR, dNLR, PLR, and SII levels were significantly lower in patients with major pathological response (MPR) versus non-MPR. On-treatment SII remained an independent predictor of MPR in multivariate logistic regression analysis. The area under the curve (AUC) of on-treatment SII for predicting MPR was 0.75 (95%CI, 0.67–0.84) in the discovery cohort. Moreover, the predictive value was further improved by combining the on-treatment SII and radiological tumor regression data, demonstrating an AUC of 0.82 (95%CI, 0.74–0.90). The predictive accuracy was validated in the external cohort. Nevertheless, the pretreatment values of these biomarkers were not correlated with pathological responses [30]. 

NLR and PLR were recently tested in patients with advanced small-cell lung cancer (SCLC) undergoing chemoimmunotherapy in a retrospective multicenter real-life study performed in China. NLR showed good predictive abilities in both terms of clinical responses and treatment-related adverse events also in this group of patients [31]. 

Numerous alternative blood-based biomarkers were investigated in recent studies. Mahiat et al. performed a study on 352 advanced stage NSCLC patients who were treated with ICIs in various settings, including 56 patients treated with chemoimmunotherapy, investigating the predictive roles of several baseline biomarkers/scores like Lung Immune Prognostic Index (LIPI), Modified Lung Immune Prognostic Index (mLIPI), Scottish Inflammatory Prognostic Score (SIPS), Advanced Lung Cancer Inflammation Index (ALI), EPSILoN, Prognostic Nutritional Index (PNI), SII, and others [32]. Among them, SII was the only biomarker also analyzed in our study, and showed a poor prognostic performance for both six-month PFS and one-year OS in the subset of patients treated with chemoimmunotherapy. The authors found that for nutrition-based indexes like ALI and PNI, higher values were associated with lower systemic inflammation and better nutritional status, as opposed to systemic inflammation indexes. In a multicenter study performed in Greece, high ALI values (>18) were significantly associated with longer OS in patients receiving ICI monotherapy [hazard ratio (HR): 0.402, *p* < 0.0001, *n* = 460], but not chemoimmunotherapy (HR: 0.624, *p* = 0.111, *n* = 212) [33]; in this last subgroup, NLR performed better, in accordance with the results of our study. 

Our study has some limitations—mainly the retrospective design, the relatively low number of patients enrolled, and the lack of external validation. In addition, only pretreatment and not on-treatment laboratory tests were retrieved, as we choose to avoid the depletion effect of chemotherapy on the blood cell populations, and its reflection on the systemic inflammation indexes under investigation. Furthermore, no data regarding the specific immune-related adverse events occurring in the patients were collected. On the other hand, our study is the first to investigate a wide number of blood test parameters and combined systemic inflammation indexes (like SIRI, SII and AISI) in patients with NSCLC who underwent chemoimmunotherapy as a first-line treatment. However, prospective studies with wider cohorts are necessary to confirm our results and better describe the potential clinical usefulness of NLR in this setting.

## 5. Conclusions

Our results showed that baseline complete blood cell count biomarkers like the absolute number of lymphocytes, the NLR, and the MLR can be useful to predict clinical outcomes in patients with advanced NSCLC treated with first-line chemoimmunotherapy. In particular, NLR showed the better AUCs in predicting both the response to treatment and the OS. Further prospective studies performed in larger cohorts are necessary to better evaluate the role and clinical applicability of such biomarkers for this specific setting.

## Figures and Tables

**Table 1:** Demographic, clinical, and blood test characteristics of the global population and after sorting for treatment response.

	Global Cohort (*n* = 62)	Responders (*n* = 47)	Non-Responders (*n* = 15)	*p*-Value
Age, years	68.5 (62.0–74.0)	66.0 (61.3–72.8)	71.0 (64.8–75.0)	0.17
Gender (M/F)	45/17	34/13	11/4	0.94
Smoking status, *n* (no/former/yes)	3/45/11	3/33/9	0/12/2	0.51
Histological type, *n* (ADK/SQ)	53/9	41/6	12/3	0.49
PD-L1, *n* (1–49%/<1%)	30/30	23/22	7/8	0.77
Stage T, *n* (T1/T2/T3/T4)	4/2/3/53	4/2/2/39	0/0/1/14	0.53
Stage N, *n* (N0/N1/N2/N3)	3/9/11/38	3/9/8/26	0/0/3/12	0.18
Deceased, *n* (yes/no)	23/37	10/35	13/2	<0.0001
Overall survival, (months)	12.1 (7.4–24.3)	14.5 (9.1–31.9)	7.5 (4.2–11.2)	0.0015
Hb (g/dL)	12.3 ± 1.7	12.6 ± 1.7	11.9 ± 1.8	0.47
RDW, (%)	14.7 (13.8–15.8)	14.7 (13.4–15.8)	14.8 (14.1–15.6)	0.53
WBC, *n* (×10^9^ L)	8.86 (7.40–11.15)	8.74 (6.91–10.72)	8.96 (7.96–13.73)	0.29
Neutrophils, *n* (×10^9^ L)	6.00 (4.10–7.60)	5.62 (3.80–7.37)	6.40 (5.59–11.95)	0.074
Lymphocytes, *n* (×10^9^ L)	1.70 (1.30–2.20)	1.80 (1.40–2.44)	1.40 (1.10–1.98)	0.10
Monocytes, *n* (×10^9^ L)	0.60 (0.50–0.80)	0.60 (0.50–0.80)	0.60 (0.50–0.80)	0.55
Platelets, *n* (×10^9^ L)	287 (253–355)	287 (254–362)	293 (247–349)	0.91
NLR	3.45 (2.18–5.47)	3.31 (2.15–4.12)	5.36 (2.78–10.82)	0.019
NMR	9.75 (7.60–11.80)	9.20 (7.45–11.20)	14.00 (8.82–21.20)	0.013
MLR	0.33 (0.23–0.53)	0.33 (0.23–0.51)	0.40 (0.21–0.55)	0.67
PLR	169 (118–246)	163 (114–244))	209 (131–248)	0.17
SII	985 (624–1838)	945 (552–1373)	1395 (929–3334)	0.025
AISI	543 (277–1072)	487 (273–955)	837 (357–1524)	0.20

ADK: adenocarcinoma; AISI: aggregate index of systemic inflammation; F: female; Hb: hemoglobin; M: male; MLR: monocyte to lymphocyte ratio; NLR: neutrophil to lymphocyte ratio; NMR: neutrophil to monocyte ratio; PD-L1: programmed death ligand 1; RDW: red cell distribution width; SII: systemic inflammation index; SQ: squamous cell cancer; WBC: white blood cells.

**Table 2:** Univariate logistic regression assessing the association between patient characteristics and treatment response.

	OR	95% CI	*p*-Value
Age, years	1.0424	0.9735 to 1.1162	0.23
Gender (M/F)	0.9510	0.2564 to 3.5275	0.94
Smoking status, *n* (no/former/yes)	1.0444	0.2906 to 3.7533	0.95
Histological type, *n* (ADK/SQ)	1.7083	0.3707 to 7.8732	0.49
PD-L1, *n* (1–49%/<1%)	1.1948	0.3706 to 3.8525	0.77
Stage T, *n* (T1/T2/T3/T4)	2.3437	0.5289 to 10.3860	0.26
Stage N, *n* (N0/N1/N2/N3)	2.7685	0.9610 to 7.9752	0.06
Hb (g/dL)	0.7851	0.5462 to 1.1286	0.19
RDW, (%)	1.1248	0.8202 to 1.5426	0.47
WBC, *n*	1.0731	0.9289 to 1.2395	0.34
Neutrophils, *n*	1.1335	0.9724 to 1.3213	0.11
Lymphocytes, *n*	0.4280	0.1620 to 1.1309	0.09
Monocytes, *n*	0.2614	0.0253 to 2.7002	0.26
Platelets, *n*	0.9987	0.9939 to 1.0036	0.60
NLR	1.2561	1.0519 to 1.4998	0.012
NMR	1.1410	1.0121 to 1.2864	0.03
MLR	1.8104	0.1299 to 25.2236	0.66
PLR	1.0018	0.9983 to 1.0053	0.32
SII	1.0002	0.9999 to 1.0005	0.27
AISI	1.0000	0.9997 to 1.0003	0.84

ADK: adenocarcinoma; AISI: aggregate index of systemic inflammation; F: female; Hb: hemoglobin; M: male; MLR: monocyte to lymphocyte ratio; NLR: neutrophil to lymphocyte ratio; NMR: neutrophil to monocyte ratio; PD-L1: programmed death ligand 1; RDW: red cell distribution width; SII: systemic inflammation index; SQ: squamous cell cancer; WBC: white blood cells.

**Table 3:** Multivariate logistic regression analysis for hematological biomarkers, in the prediction of treatment response.

	Model 1			Model 2		
	aOR	95% CI	*p*-Value	aOR	95% CI	*p*-Value
NLR	1.3210	1.0648 to 1.6387	0.01	1.8300	1.1236 to 2.9806	0.02
NMR	1.1585	1.0070 to 1.3328	0.04	1.1698	1.0019 to 1.3657	0.047

Model 1: correction performed with age, gender smoking status, histological type, PD-L1. Model 2: correction performed with age, gender smoking status, stage T and stage N. NLR: neutrophil to lymphocyte ratio; NMR: neutrophil to monocyte ratio.

**Table 4:** Demographic, clinical, and blood test characteristics of the global population and after sorting for survival.

	Global Cohort (*n* = 60)	Survivors (*n* = 37)	Non-Survivors (*n* = 23)	*p*-Value
Age, years	68.0 (62.0–73.5)	66.0 (62.0–73.3)	70.0 (61.5–74.5)	0.37
Gender (M/F)	44/16	27/10	17/6	0.94
Smoking status, *n* (no/former/yes)	3/43/11	3/25/7	0/18/4	0.35
Histological type, *n* (ADK/SQ)	51/9	33/4	18/5	0.25
PD-L1, *n* (1–49%/<1%)	29/29	18/17	11/12	0.79
Stage T, *n* (T1/T2/T3/T4)	4/2/3/51	4/2/2/29	0/0/1/22	0.23
Stage N, *n* (N0/N1/N2/N3)	3/8/10/38	3/7/5/21	0/1/5/17	0.15
Hb (g/dL)	12.3 ± 1.7	12.6 ± 1.8	12.2 ± 1.7	0.43
RDW, (%)	14.6 (13.6–15.7)	14.4 (13.3–15.8)	14.8 (14.1–15.6)	0.51
WBC, *n* (×10^9^ L)	8.94 (7.41–11.53)	8.26 (6.36–9.92)	9.69 (7.97–14.03)	0.026
Neutrophils, *n* (×10^9^ L)	6.00 (4.10–7.81)	5.30 (3.48–7.03)	7.00 (6.00–11.95)	0.001
Lymphocytes, *n* (×10^9^ L)	1.70 (1.30–2.23)	1.80 (1.40–2.50)	1.50 (1.13–2.00)	0.14
Monocytes, *n* (×10^9^ L)	0.60 (0.50–0.80)	0.60 (0.50–0.80)	0.80 (0.50–0.80)	0.32
Platelets, *n* (×10^9^ L)	287 (252–353)	270 (238–336)	314 (281–407)	0.052
NLR	3.45 (2.20–5.42)	2.94 (1.92–3.88)	4.56 (3.07–9.49)	0.012
NMR	9.60 (7.60–11.75)	9.00 (7.08–11.05)	10.40 (8.80–18.18)	0.007
MLR	0.34 (0.24–0.54)	0.33 (0.24–0.41)	0.43 (0.24–0.58)	0.16
PLR	169 (119–246)	136 (107–201)	220 (145–273)	0.016
SII	985 (626–1709)	849 (488–1081)	1493 (1000–2578)	0.0004
AISI	594 (279–1168)	351 (256–794)	1016 (470–1836)	0.006

ADK: adenocarcinoma; AISI: aggregate index of systemic inflammation; F: female; Hb: hemoglobin; M: male; MLR: monocyte to lymphocyte ratio; NLR: neutrophil to lymphocyte ratio; NMR: neutrophil to monocyte ratio; PD-L1: programmed death ligand 1; RDW: red cell distribution width; SII: systemic inflammation index; SQ: squamous cell cancer; WBC: white blood cells.

**Table 5:** Univariate logistic regression assessing the association between patient characteristics and survival.

	OR	95% CI	*p*-Value
Age, years	1.0268	0.9706 to 1.0863	0.36
Gender (M/F)	0.9529	0.2928 to 3.1016	0.94
Smoking status, *n* (no/former/yes)	1.3482	0.4379 to 4.1505	0.60
Histological type, *n* (ADK/SQ)	2.2917	0.5458 to 9.6219	0.26
PD-L1, *n* (1–49%/<1%)	1.1551	0.4030 to 3.3107	0.79
Stage T, *n* (T1/T2/T3/T4)	3.4877	0.6931 to 17.5496	0.13
Stage N, *n* (N0/N1/N2/N3)	2.0009	0.9653 to 4.1472	0.06
Hb (g/dL)	0.8804	0.6468 to 1.1982	0.42
RDW, (%)	1.1224	0.8410 to 1.4978	0.43
WBC, *n*	1.2202	1.0339 to 1.4400	0.019
Neutrophils, *n*	1.2916	1.0692 to 1.5604	0.008
Lymphocytes, *n*	0.5819	0.2719 to 1.2454	0.16
Monocytes, *n*	1.7055	0.2449 to 11.8784	0.59
Platelets, *n*	1.0014	0.9977 to 1.0052	0.45
NLR	1.3601	1.0949 to 1.6896	0.005
NMR	1.2159	1.0396 to 1.4221	0.015
MLR	5.6613	0.4789 to 66.9198	0.17
PLR	1.0023	0.9988 to 1.0058	0.19
SII	1.0004	1.0000 to 1.0007	0.054
AISI	1.0002	0.9999 to 1.0005	0.20

ADK: adenocarcinoma; AISI: aggregate index of systemic inflammation; F: female; Hb: hemoglobin; M: male; MLR: monocyte to lymphocyte ratio; NLR: neutrophil to lymphocyte ratio; NMR: neutrophil to monocyte ratio; PD-L1: programmed death ligand 1; RDW: red cell distribution width; SII: systemic inflammation index; SQ: squamous cell cancer; WBC: white blood cells.

**Table 6:** Multivariate logistic regression analysis for hematological biomarkers in the prediction of mortality.

	Model 1			Model 2		
	aOR	95% CI	*p*-Value	aOR	95% CI	*p*-Value
WBC	1.2596	1.0458 to 1.5171	0.015	1.2475	1.0317 to 1.5084	0.023
Neutrophils	1.3112	1.0698 to 1.6071	0.009	1.2990	1.0527 to 1.6028	0.015
NLR	1.3498	1.0758 to 1.6936	0.01	1.3489	1.0632 to 1.7114	0.014
NMR	1.2502	1.0311 to 1.5158	0.02	1.5685	1.0901 to 2.2568	0.015

Model 1: correction performed with age, gender smoking status, histological type, PD-L1. Model 2: correction performed with age, gender smoking status, stage T and stage N. NLR: neutrophil to lymphocyte ratio; NMR: neutrophil to monocyte ratio; WBC: white blood cells.

**Table 7:** Diagnostic performances of hematological biomarkers in the prediction of mortality.

	AUC	95% CI	*p*-Value	Cut-Off	Sensitivity	Specificity
WBC	0.672	0.539 to 0.788	0.017	>11.98	39	89
Neutrophils	0.746	0.617 to 0.849	0.0001	>5.7	83	65
NLR	0.749	0.620 to 0.852	0.0001	>4.0	61	81
NMR	0.707	0.576 to 0.818	0.0038	>11.8	48	92

NLR: neutrophil to lymphocyte ratio; NMR: neutrophil to monocyte ratio; WBC: white blood cells.

**Table 8:** Multivariate Cox regression analysis for hematological biomarkers in the prediction mortality.

	Model 1			Model 2		
	aHR	95% CI	*p*-Value	aHR	95% CI	*p*-Value
WBC	1.1966	1.0443 to 1.3711	0.01	1.1191	0.9944 to 1.2594	0.062
Neutrophils	1.2297	1.0745 to 1.4074	0.003	1.1480	1.0162 to 1.2970	0.027
NLR	1.3016	1.1267 to 1.5037	0.003	1.2141	1.0666 to 1.3819	0.003
NMR	1.0217	1.0056 to 1.0380	0.008	1.0174	1.0027 to 1.0324	0.021

Model 1: correction performed with age, gender smoking status, histological type, PD-L1. Model 2: correction performed with age, gender smoking status, stage T and stage N. NLR: neutrophil to lymphocyte ratio; NMR: neutrophil to monocyte ratio; WBC: white blood cells.

## Data Availability

Data are available by the corresponding author upon reasonable request.
